# Syringing has limited reliability in differentiating nasolacrimal duct stenosis from functional delay

**DOI:** 10.1007/s00417-022-05654-1

**Published:** 2022-04-23

**Authors:** Yinon Shapira, Valerie Juniat, Carmelo Macri, Dinesh Selva

**Affiliations:** 1 Discipline of Ophthalmology and Visual Science, University of Adelaide, Adelaide, South Australia Australia; 2 Department of Ophthalmology, Royal Adelaide Hospital and South Australian Institute of Ophthalmology, Adelaide, South Australia Australia

**Keywords:** Epiphora, Lacrimal syringing, Irrigation, Lacrimal scintigraphy, Dacryoscintigraphy, Dacryocystography, Nasolacrimal duct, Stenosis, Delay, Functional

## Abstract

**Purpose:**

To elucidate the role of syringing in assessing nasolacrimal duct (NLD) stenosis and non-anatomical functional NLD delay.

**Methods:**

Consecutive adult patients with epiphora attending a tertiary lacrimal clinic from June 2011 to March 2021 were reviewed. Cases with evidence of canalicular stenosis or other identifiable causes of epiphora were excluded. Following syringing, patients were investigated with dacryocystography (DCG) and dacryoscintigraphy (DSG). The sensitivity and specificity of syringing were evaluated using the combined findings on DCG and DSG.

**Results:**

A total of 289 symptomatic lacrimal systems (197 patients; mean age 65.5 ± 14.9 years, 66% females) were included. More than one-third of cases with both normal DCG and DSG were noted to have some degree of reflux on syringing (specificity = 65.1%, 95% CI 50.2–77.6%). The sensitivities were considerably low for NLD stenosis (i.e., stenosis on DCG and delay on DSG) and for functional NLD delay (i.e., normal DCG and delay on DSG), of which 43.7% (95% CI 32.2–55.9%) and 54.3% (95% CI 45.7–62.7%) had full patency on syringing, respectively (*p* = 0.17).

**Conclusions:**

Full patency on syringing was unreliable for ruling out NLD stenosis and functional delay. Furthermore, a positive syringing may be associated with functional NLD delay and cannot reliably differentiate it from stenosis.
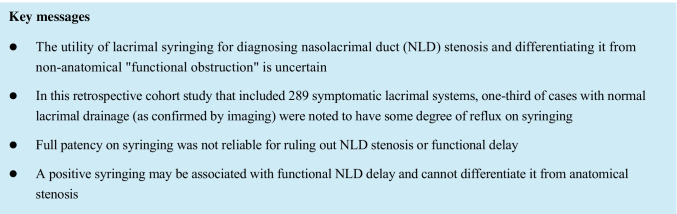

## Introduction

Lacrimal syringing is commonly used to investigate epiphora [1, 2], but there is limited data on its sensitivity and specificity [3–6]. The utility of this test for diagnosing nasolacrimal duct (NLD) stenosis and non-anatomical “functional obstruction” is particularly uncertain [7, 8].

Lacrimal investigations such as dacryocystography (DCG) and dacryoscintigraphy (DSG) can be used to evaluate the anatomy and function of the lacrimal drainage system, respectively [7, 9]. However, these tests require time and resources and are not always utilized [10].

The current study aimed to determine the sensitivity and specificity of syringing in NLD drainage impairment (anatomical obstruction, stenosis, or non-anatomical functional delay), as diagnosed by combined DCG and DSG findings.

## Methods

Data were collected retrospectively from consecutive adult patients with epiphora attending the Royal Adelaide Hospital lacrimal clinic from June 2011 to March 2021. The study received Institutional Review Board (IRB) approval and adhered to the tenets of the Declaration of Helsinki.

Patients were excluded if they had puncto-canalicular obstruction/stenosis, eyelid malposition/paralysis, potential causes of reflex tearing, acute dacryocystitis, refluxable mucoceles, or previous lacrimal surgery. All patients underwent syringing as part of their clinical assessment. Trained, experienced oculoplastics surgeons carried out syringing. A lacrimal cannula on a 2-ml syringe was inserted 1–2 mm vertically through the punctum. The lid was distracted laterally by the operator’s finger and kept under tension. The lacrimal cannula was slowly advanced through the canaliculus until it reached a hard or soft stop. The cannula was held in place while syringing was performed under minimal pressure. The degree of reflux was recorded. As all suspected cases of canalicular or common canalicular obstruction (or stenosis) were excluded from this study (focusing on NLD impairment), the site of reflux was always the opposite punctum.

Patients were subsequently investigated with DCG and DSG performed by trained radiologists. An experienced oculoplastic surgeon assessed the imaging studies.

### DCG technique

DCGs were performed with patients in the supine position. A drop of topical anesthetic (1% tetracaine hydrochloride) was instilled into the inferior conjunctival fornix of both eyes. The punctum was cannulated with a 27-gauge lacrimal cannula. Baseline X-ray images were taken, followed by real-time imaging during injection of contrast (iopromide, Ultravist® 370; Bayer HealthCare Pharmaceuticals, Germany) through the cannula. This allowed digital subtraction of the pre-contrast image from post-contrast images. “NLD stenosis” was defined as having a duct diameter of less than that of the width of the lacrimal cannula tip on the X-ray image (27 gauge, 0.4 mm external diameter) but with patency [11]. “NLD obstruction” was defined as no patency through the duct on DCG.

### DSG technique

A 10-ml drop of technetium-99 m pertechnetate was placed into both eyes with the patient sitting upright in front of a gamma camera. One-minute sequential images were taken over 45 min. At the end of the serial scanning, if the tracer has not sufficiently progressed to reach the nasal cavity in any eye, the participant was asked to clear their nasal passages, and a lacrimal massage was applied to both eyes. Another 45 min of a 1-min sequential scan was then subsequently acquired. The appearance of tracer at the lacrimal sac, lacrimal duct, or nasal cavity was recorded. Five minutes was used as the cut-off time-point to qualitatively determine normal versus post-sac (NLD) delay based on end-tracer location [11, 12].

### Data analysis

#### Defining NLD obstruction, stenosis, and functional delay

NLD drainage assessment was based on the combined DCG and DSG findings and categorized into four categories: Normal (DCG = normal, DSG = normal), NLD obstruction (NLDO) (DCG = obstruction, DSG = delay), NLD stenosis (NLDS) (DCG = stenosis, DSG = delay), or functional NLD delay (FNLDD) (DCG = normal, DSG = delay).

#### Defining patency on syringing

The grading of the proportion reflux on syringing was utilized to define patency. In order to analyze the degree of reflux as a differentiating feature, the cut-off for defining a “positive” (i.e., abnormal) syringing was set according to four different criteria and analyzed separately: “full patency” (0% reflux, defining a “negative syringing” versus > 0% reflux, defining a “positive syringing”); “80% (partial) patency” (< 20% reflux versus ≥ 20% reflux); “50% (partial) patency” (< 50% reflux versus ≥ 50% reflux); or “no patency” (< 100% reflux, defining a negative syringing versus 100% reflux, defining a positive syringing).

#### Sensitivity and specificity analysis

Syringing specificity values correspond to the proportion of lacrimal systems with normal NLD drainage (DCG and DSG) and negative in the syringing test (true negatives).

The syringing sensitivity values correspond to the proportion of lacrimal systems with NLD drainage impairment (DCG and/or DSG) and positive in the syringing (true positives).

#### Statistical analysis

Data were analyzed by the StatSoft Statistica software, version 10 (StatSoft, OK, USA). Proportions were compared by the chi-square test. A two-sided *p*-value of less than 0.05 was considered significant.

## Results

A total of 289 symptomatic lacrimal systems of 197 patients met the inclusion criteria: 85 patients had unilateral symptoms, 73 had bilateral and equal symptoms, and 29 patients had bilateral and asymmetrical symptoms (as per patient-reported symptoms). The mean age was 65.5 ± 14.9 years (range 18–94 years), and 130 (66%) were females.

Overall, 43 (14.9%) lacrimal systems had normal drainage on DCG and DSG, 55 (19%) had NLDO, 64 (22.1%) had NLDS, and 127 (43.9%) had FNLDO.

Table 1 presents the reliability (i.e., sensitivity and specificity) of syringing for detecting NLDO, NLDS, and FNLDD based on combined DCG and DSG findings.

**Table 1:**
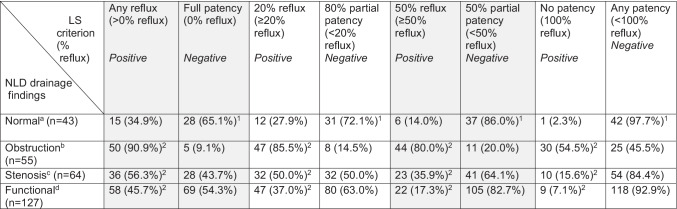
The reliability of syringing in detecting NLD impaired drainage based on combined DCG and DSG findings in watery eyes, according to four cut-off criteria for defining a positive syringing

*NLD* nasolacrimal duct, *DCG* dacryocystography, *DSG* dacryoscintigraphy, *LS* lacrimal syringing

^a^DCG and DSG normal

^b^DCG obstruction and DSG delay

^c^DCG stenosis and DSG delay

^d^DCG normal and DSG delay

^e^Proportions correspond to the syringing test specificity values (true negatives)

^f^Proportions correspond to the syringing test sensitivity values (true positives)

### The specificity of syringing in normal NLD drainage (on DCG and DSG)

Of the 43 lacrimal systems demonstrating normal drainage on DCG and DSG, 65.1% (95% CI 50.2–77.6%) were fully patent (true negatives) on syringing. This denotes a moderate specificity when defining an abnormal syringing as any reflux, as more than a third of cases were falsely positive using this cut-off.

The specificity improved when using syringing less stringent, “partial patency” (cut-off) criteria (Table 1). Namely, the syringing specificity was 72.1% (95% CI 57.3–83.3%) at < 20% reflux and was 86% (95% CI 72.7–93.5%) at < 50% reflux. When syringing showed any degree of patency (< 100% reflux), the specificity was 97.7% (95% CI 87.9–99.6%). In other words, no patency on syringing was noted only in one normal lacrimal system (2.3% false-positive rate).

### The sensitivity of syringing in cases with NLD drainage impairment (on DCG and/or DSG)

NLDO manifested as (> 0%) reflux on syringing in 90.9% (95% CI 80.4–96.1%) of cases, representing a high sensitivity (true positives) at this syringing test cut-off. The sensitivity slightly declined in syringing’s partial patency (cut-off) criteria; however, it remained high (Table 1). Namely, the syringing sensitivity was 85.5% (95% CI 73.8–92.4%) at ≥ 20% reflux and was 80% (95% CI 67.6–88.5%) at ≥ 50% reflux. The sensitivity was moderate (54.5%, 95% CI 41.5–67.0%) at no patency on syringing (100% reflux). In other words, close to half (45.5%) of imaging-confirmed NLDO cases had some patency on syringing.

In cases of NLDS, the sensitivity of syringing was moderate (56.3%, 95% CI 44.1–67.7%) at > 0% reflux and was low across the less stringent cut-off definitions (Table 1): 50% (95% CI 38.1–61.9%) at ≥ 20% reflux, 35.9% (95% CI 25.3–48.2%) at ≥ 50% reflux, and 15.6% (95% CI 8.7–26.4%) at 100% reflux. The syringing sensitivity values in NLDS were significantly lower than the respective sensitivities in NLDO (*p* < 0.0001 for all).

The sensitivity of syringing in FNLDD was low across all cut-off definitions (Table 1): 45.7% (95% CI 37.3–54.3%) at > 0% reflux (*p* = 0.17 compared to the sensitivity in NLDS), 37.0% (95% CI 29.1–45.7%) at ≥ 20% reflux (*p* = 0.09 compared to NLDS), 17.3% (95% CI 11.7–24.8%) at ≥ 50% reflux (*p* = 0.004 compared to NLDS), and 7.1% (95% CI 3.8–12.9%) at 100% reflux (*p* = 0.06 compared to NLDS).

## Discussion

Our results firstly show that in a cohort of patients with epiphora where alternate causes have been clinically excluded, a negative syringing (100% patency) failed to detect NLDS in 44% and FNLDD in 54% of cases. These results confirm that lacrimal syringing is unreliable for ruling out NLD stenosis or functional delay. Furthermore, full patency (or a negative syringing) is used by many to be a criterion for the diagnosis of non-anatomical FNLDD [13–16]. Our results suggest that significant NLD stenosis could likewise be found to be fully patent on syringing and in a similarly high proportion of cases. They further show that reflux on syringing may be found in close to half of FNLDD cases and cannot be used to differentiate it from NLDS.

The results also suggest that while any reflux may indicate pathology, it is also found in “normal” systems as defined by imaging. That is, the specificity of syringing was found to be limited, as approximately one-third of the cases that had normal NLD drainage on imaging were noted to have some degree of reflux on syringing. On the other hand, no patency on syringing was highly specific, with only one false-positive case. Taken together, no patency on syringing would suggest NLD impaired drainage with high confidence (98%), while partial (but not full) patency would suggest impaired drainage with lower confidence (65%).

The detection of NLD stenosis and non-anatomical functional delay is particularly challenging in the clinical setting, and the role of syringing in these cases has not been clearly characterized to date [7]. Syringing is the most frequently used, sometimes stand-alone test in clinical practice [1, 2]. On the other hand, lacrimal imaging studies are used infrequently [10]. Clinicians could thus benefit from knowing the strengths and limitations of syringing in the context of NLD drainage impairment, especially when patency (or partial patency) is demonstrated. This may guide consideration of further investigation and have implications for consenting patients regarding intervention success rates.

Whereas other studies have analyzed the relationship of syringing to DCG and DSG findings separately, to our knowledge, this study is the first to correlate lacrimal syringing to the combined (and complementary) findings on DCG and DSG [4–6, 12, 15]. The combination of these imaging studies currently provides the closest “gold standard” to diagnose the specific type and degree of NLD drainage impairment (before intraoperative confirmation). Namely, it could differentiate complete anatomical obstruction (NLDO), from partial obstruction (NLDS), from non-anatomical “functional” delay (FNLDD) [7, 8]. FNLDD was historically defined as “incomplete blockage” based on negative Jones 1 and a positive Jones 2 test [17, 18]. Nonetheless, this methodology probably incorporates stenosis and non-anatomical (functional) delay in the same cohort [8]. Furthermore, the Jones test is less commonly used in the lacrimal clinic than syringing [1].

The correlation between syringing and lacrimal imaging (DCG or DSG, separately) has been previously investigated; however, various definitions for full patency or a normal syringing were used. Some authors defined a fully patent syringing as less than 20% reflux [4, 12], others as 0% reflux [3, 5], while some did not clearly state their definition of normal syringing [6]. Our results suggest that any reflux should be considered a positive test, and the utility of less stringent criteria (such as > 20% reflux) is probably limited due to significantly diminished sensitivities. Furthermore, these previous studies did not stratify the results by the site of imaging abnormality (presac or postsac), whereas the current analysis focused on post sac (NLD) impairment.

These methodological discrepancies notwithstanding, our reported syringing general specificity (65%) and sensitivity for NLDO (91%) compare favorably with the figures reported by Nixon et al. [6] based on their comparison to DCG (53% and 86%, respectively). Our reported sensitivities for FNLDD (46%) and NLDS (56%) are similar to those reported by Kim et al. [5] In their study, reflux on syringing had a sensitivity of 50% when the DSG showed delay. Arguably, their results based only on DSG capture both stenosis and functional delay, thus falling within the range of our reported figures.

One previous study compared syringing to DCG and DSG separately [4]. The authors defined less than 20% reflux on syringing as a “freely patent” result. Peter and Pearson’s [4] study yielded a syringing sensitivity of 28.6% for anatomic abnormality on DCG and 25% for delay on DSG. While in the current analysis, the same cut-off of syringing (20% reflux) yielded higher sensitivity in NLDS (50%) and FNLDD (37%), both studies underscore the limitation of syringing in detecting these impairments.

The strengths of this study include a large number of patients and the use of comprehensive imaging (DCG and DSG), allowing analysis of homogenous etiologies of drainage impairment (NLDO, NDLS, NLDD). This study’s limitations firstly include its retrospective nature. Another limitation is using DCG and DSG as the diagnostic reference, as these modalities are themselves confined by investigative sensitivity and specificity constraints [6, 19, 20]. Nevertheless, combining both modalities may increase the sensitivity to 98% [15], and they are considered complimentary [8, 21]. Noteworthy, dacryoendoscopy can directly visualize an obstruction’s degree, level, and nature and perhaps be a better diagnostic gold standard [22, 23]. Nonetheless, it is a lacrimal procedure, often with simultaneous treatment of obstruction, and requires injection of local anesthesia (possibly with the addition of sedation in selected cases). It also entails special equipment (dacryoendoscope) that is not widely available yet.

Last, since syringing is a crude test, inter-tester variations in the exact pressure and subjective estimation of reflux (proportions) are possible. Nevertheless, in the current study, oculoplastic surgeons performed syringing, all trained under the last author (DS), who supervised and validated the technique. Thus, adherence to the same testing standards could be assumed for the entire study period. Furthermore, we categorized the estimated reflux to four cut-offs (100%, < 50%, < 20%, 0%) which were scrutinized separately. Thus, grouping into categories (ranges) and not relying on “exact” proportions should minimize inter-tester bias.

In conclusion, full patency on syringing was unreliable for ruling out NLD stenosis or functional delay. Hence, in patients with troublesome epiphora who are fully patent on syringing, imaging may determine the presence of NLD impairment and exclude those with normal systems which would be unlikely to benefit from intervention.

We believe that based on the results, any reflux should be considered an abnormal test in the context of epiphora. Finally, a positive syringing may be associated with functional NLD delay and cannot reliably differentiate it from stenosis, and this may have implications for consenting patients regarding success rates of intervention.
